# Cutaneous manifestations of diabetes mellitus: a narrative review

**DOI:** 10.31744/einstein_journal/2025RW1193

**Published:** 2025-03-10

**Authors:** Maria Carolina Mendes de Oliveira Abate, Priscila Maria Teixeira Aroucha, Diego Vanderlei Medeiros da Nóbrega, Iara Patrícia Moura Rocha, Sofia Duarte Soares, Anita Andrade Reis, Isabella Cristina Paliares, Fernando de Mello Almada Giuffrida, Sergio Atala Dib, André Fernandes Reis, Joao Roberto de Sa

**Affiliations:** 1 Centro de Endocrinologia e Diabetes Escola Paulista de Medicina Universidade Federal de São Paulo São Paulo SP Brazil Division of Endocrinology, Centro de Endocrinologia e Diabetes, Escola Paulista de Medicina, Universidade Federal de São Paulo, São Paulo, SP, Brazil.; 2 Department of Dermatology Escola Paulista de Medicina Universidade Federal de São Paulo São Paulo SP Brazil Department of Dermatology, Escola Paulista de Medicina, Universidade Federal de São Paulo, São Paulo, SP, Brazil.; 3 Department of Life Sciences Universidade do Estado da Bahia Salvador BA Brazil Department of Life Sciences, Universidade do Estado da Bahia, Salvador, BA, Brazil.; 4 Division of Endocrinology and Metabolic Disease Centro Universitário FMABC Santo André SP Brazil Division of Endocrinology and Metabolic Disease, Centro Universitário FMABC, Santo André, SP, Brazil.

**Keywords:** Diabetes mellitus, Skin diseases, Diabetes mellitus, type 1, Diabetes mellitus, type 2, Diagnosis, differential

## Abstract

Diabetes mellitus is a highly prevalent human endocrine disorder. Skin lesions are reported in approximately one-third of all diabetes mellitus patients. The clinical presentation and frequency vary according to the subtype of diabetes mellitus, metabolic control, and clinical course, with certain skin diseases occurring before diagnosing hyperglycemia. In this regard, the correct definition of cutaneous manifestations associated with diabetes mellitus can help define the etiology of hyperglycemia as well as the need to optimize glycemic control. In this narrative review, the most common cutaneous diseases observed in diabetes mellitus are discussed, including pruritus, acanthosis nigricans, necrobiosis lipoidica, bullosis diabeticorum, scleroderma diabeticorum, granuloma annulare, diabetic dermopathy, skin reactions due to device use, diabetic foot ulcers, recurrent cutaneous infections in diabetes mellitus and other dermatoses associated with hyperglycemia. The epidemiology, pathophysiology, differential diagnosis, and treatment of this disease are discussed. Therefore, knowledge and recognition of the most common dermatological lesions in patients with diabetes mellitus are essential for both endocrinologists and primary care physicians.

## INTRODUCTION

*Diabetes Mellitus* (DM) is an epidemic disease with an increasing number of patients, mainly in developing countries. *Diabetes Mellitus* constitutes a global health problem that has a significant impact on health systems.^1^ Elevated glucose levels may affect the skin, leading to non-infectious and infectious dermatological conditions and symptoms. Approximately one-third of all DM patients exhibit cutaneous clinical manifestations during the disease course.^2^ In addition, certain antidiabetic therapies may occasionally induce skin complications.^3^ Several other factors underlie the importance of correctly diagnosing skin lesions associated with DM: the potential psychological burden caused by the stigmatization of visible skin lesions; early diagnosis of DM based on cutaneous manifestations; association of specific lesions with a particular subtype of DM, helping to correctly define its etiology; and association of skin lesions with poorly controlled disease in those already diagnosed with DM, indicating the need to optimize metabolic control, thereby preventing diabetic complications.^4^

This review discusses the most common cutaneous diseases associated with DM. The epidemiology, pathophysiology, differential diagnosis, and treatment of this disease are discussed. PubMed was searched for publications on the subject using the following terms: DM and skin disease, type 1 diabetes and skin disease, type 2 diabetes and skin disease, and cutaneous manifestations of DM. The search was performed on September 1, 2024. The results of relevant and recent papers published in English were manually screened. References from selected publications were used when necessary.

### PREVALENCE, INCIDENCE, AND PATHOGENESIS OF MOST COMMON FORMS OF *DIABETES MELLITUS*

An estimated 537 million adults live with DM worldwide.^5^ Considering that approximately one-third of people develop skin lesions,^2^ 179 million people are affected by secondary skin changes due to or related to DM.

*Diabetes mellitus* is a heterogeneous group of disorders involving carbohydrate metabolism that can be classified into several clinical categories. Type 1 DM (T1DM) and type 2 DM (T2DM) are the most prevalent types. Correct classification and understanding related pathophysiological mechanisms are clinically relevant because they can determine personalized therapy and prognosis. T1DM is caused by autoimmune destruction of pancreatic beta cells, resulting in near-absolute insulin deficiency.^6^ Autoimmunity is triggered by environmental stimuli (such as viral infections) in the context of an underlying genetic predisposition. T-lymphocyte-mediated destruction of beta cells results in antigen exposure, giving rise to antibodies targeting specific antigens such as insulin, glutamic acid decarboxylase (GAD), insulinoma-associated protein 2 (IA-2), or zinc transporter 8 (ZnT8). Antibody titers are related to both the intensity and progression of autoimmunity as well as the severity of insulin deficiency; therefore, they can have both diagnostic and prognostic value. The characterization of the underlying pathophysiology is less accurate in T2DM. Environmental factors such as obesity and a sedentary lifestyle are involved in its evolution, as well as heredity, in the context of beta-cell dysfunction, which is less well-defined than in T1DM.^6^ A coexistence of deficient insulin secretion associated with different degrees of insulin resistance exist, often associated with other components of metabolic syndrome, such as arterial hypertension, dyslipidemia, and low-grade inflammation.^6^ Other rare monogenetic forms (such as MODY) have been described in recent decades and are mainly related to genetic insulin secretion defects.^7^ Chronic hyperglycemia, regardless of the type of DM, is associated with the development and progression of chronic complications (*i.e*., diabetic retinopathy, nephropathy, and neuropathy).^6^

### PATHOGENESIS OF CUTANEOUS MANIFESTATIONS IN *DIABETES MELLITUS*

Hyperglycemia leads to direct cell damage in the dermis, epidermis, keratinocytes, and fibroblasts, as well as indirect impairment through advanced glycation end products (AGEs). Advanced glycation end-products may induce free radical production, causing oxidative stress. In addition, the mechanisms of vasodilation and vascular supply to the skin are compromised by the inhibition of the nitric oxide (NO) pathway.^8,9^ Moreover, the sorbitol pathway was upregulated, thereby increasing oxidative stress.^10^ The binding of AGEs with their specific receptor promotes nuclear factor kappa-light-chain-enhancer of activated B cells (NF-κB) activation and consequent production of proinflammatory cytokines,^9,11^ amplifying the inflammatory response, epithelial damage, and skin lesions. A few cutaneous manifestations may be indicative of T1DM or T2DM, depending on their pathogenesis (Table 1).

**Table 1:** Association between cutaneous disorders and diabetes mellitus

Condition	DM type association	Clinical presentation	Differential diagnosis	Treatment
Pruritus	T1DM=T2DM	Generalized scratches	Cutaneous xerosis, metabolic causes, paraneoplastic	Control underlying cause. Oral antihistamines. Tricyclic antidepressants^(^^12^^-^^14^^)^
Acanthosis nigricans	T2DM>T1DM	Velvety, hyperchromic plaques	Malignant acanthosis nigricans	Control underlying cause, retinoids, octreotide^(^^17^^)^
Necrobiosis lipoidica	T1DM>T2DM; HNF1A-MODY	Ovoid plaques with yellow-brown atrophic center	Sarcoidosis, squamous cell carcinoma, xanthomas	Topical and intralesional steroids, control underlying cause^(^^22^^-^^24^^)^
Bullous diabeticorum	T1DM>T2DM	Tense bullae on trauma-associated sites	Bullous pemphigoid, epidermolysis bullosa acquisita	Conservative. Drainage. Close observation for secondary infection^(^^28^^)^
Scleredema diabeticorum	T2DM>T1DM	Skin thickening and induration, peau d'orange appearance	Mycosis fungoides, scleroderma, poikiloderma	Generally unsatisfactory. PUVA^(^^32^^)^
Granuloma annulare	T1DM>T2DM; HNF1A-MODY	Oval plaques with well-demarcated edges in a centrifugal pattern	Psoriasis, sarcoidosis, leprosy	Control underlying cause. Topical steroids. UVB-NB^(^^24^^,^^32^^)^
Diabetic dermopathy	T1DM=T2DM	Small, brown, round to ovoid atrophic depressions	Stasis eczema, Necrobiosis lipoidica, lichen planus, amyloidotic lichen	None recommended^(^^50^^)^
Skin reactions due to device use	T1DM> T2DM	Erythema, lipodystrophy (specific to CSII use), subcutaneous nodules, scars, and pruritus	Contact dermatitis, bacterial infection	Avoid broken skin; rotate through multiple sites. Symptomatic relief medications. Antibiotics, if necessary^(^^55^^)^
Diabetic foot ulcers	T1DM=T2DM	Foot ulcers, erythema, local warmth, purulent discharge in case of infection	Arterial and venous ulcers, Squamous cell carcinoma.	Debridement,^(^^58^^)^ wound dressings,^(^^58^^)^ Hyperbaric and topic therapy,^(^^66^^)^ antimicrobial therapy,^(^^58^^)^ LeucoPatch system^(^^67^^)^
Candidiasis	T1DM=T2DM	Macerated erythematous plaques	Contact dermatitis, tinea cruris	Topical and/or oral antifungals^(^^68^^)^
Eruptive xanthomas	T1DM <T2DM	Cup-shaped, isolated, or confluent yellow papules with an erythematous base	Basal cell carcinoma, histiocytosis, granuloma annulare	Surgical removal, serum triglycerides control^(^^74^^)^

T1DM: type 1 diabetes mellitus; T2DM: type 2 diabetes mellitus; PUVA: phototherapy treatment with psoralen and UVA radiation; UVB-NB: phototherapy treatment with UVB narrowband radiation; CSII: continuous subcutaneous insulin infusion.

### MOST COMMON SKIN ABNORMALITIES IN *DIABETES MELLITUS*

### Pruritus

Chronic pruritus is one of the most common complaints in patients with DM and is closely associated with xerosis cutis.^12,13^ Up to 50% of patients with DM experience itching, impairing their quality of life.^13^ Furthermore, diabetic polyneuropathy with sweating dysfunction may play a role in the pathogenesis of diabetic pruritus.^14^ The fundamental therapeutic measures are the control of glucose levels and skin hydration with emollients, such as urea-containing moisturizers, amended by topical antipruritics, such as polidocanol or menthol.^15^ In more severe and extensive cases, therapy with topical corticosteroids, such as mometasone furoate or betamethasone dipropionate, or intralesionally injected corticosteroids such as triamcinolone acetonide, may be necessary.^16^ Oral antihistamines (promethazine hydrochloride at night) or tricyclic antidepressants (doxepin at night) can be administered in refractory cases. Nonsedating antihistamines and selective serotonin reuptake inhibitors (SSRI) antidepressants may reduce pruritus. Ultraviolet phototherapy can be considered a treatment option for refractory diseases.^16^

#### Acanthosis nigricans

**Figure 1:**
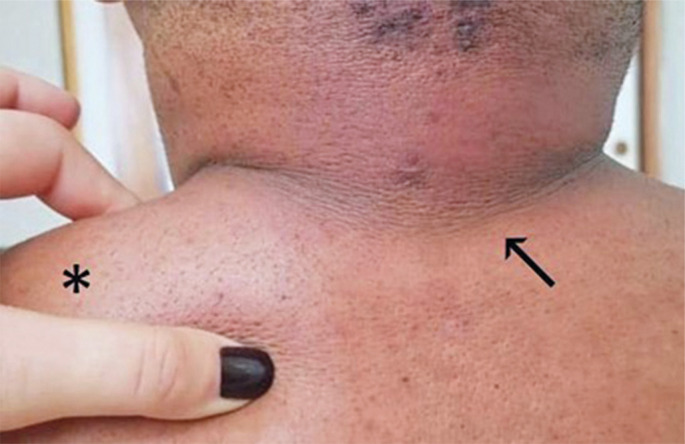
Hyperchromic velvety plaques in folding areas compatible with acanthosis nigricans (arrow) and diffuse erythema and thickening of the skin, shown by the examiner’s hand, on the upper body compatible with scleredema diabeticorum (asterisk)

Acanthosis nigricans (AN) is another common dermatological manifestation of endocrinopathies and metabolic diseases associated with obesity and hyperinsulinemia and is considered a significant clinical indicator of insulin resistance; it is less commonly a paraneoplastic manifestation of gastrointestinal adenocarcinoma and other tumors (lung, breast, and ovarian cancer).^17^ Acanthosis nigricans is characterized by symmetric, dark, thickened, and velvety plaques that are mostly distributed on the neck, intertriginous areas, and flexures^17,18^ (Figure 1).

The prevalence of AN is increasing due to the increased incidence of T2DM and obesity. In a multicenter cross-sectional study of 1730 persons aged 7–65 years presenting with primary care, AN was present in 19.4% of the patients; additionally, patients with AN were twice as likely to have T2DM as those without AN (35.4% *versus* 17.6%, p<0.01).^19^ The pathophysiology of AN remains unclear. Insulin and insulin growth factors (IGFs) play major roles. At high concentrations, insulin activates the IGF-1 receptors observed in keratinocytes and fibroblasts, thereby inducing AN.^20^

Local treatments for AN are usually unsatisfactory, and correction of contributing factors, such as obesity, should be the target. The use of medications that reduce insulin resistance and, consequently, hyperinsulinemia, such as metformin and thiazolidinediones, can ameliorate AN, although the degree of improvement is variable and complete remission does not always occur. Octreotide has been observed to improve AN, presumably via the inhibition of glucagon, growth hormone, and insulin release.^17^

#### Necrobiosis lipoidica

**Figure 2:**
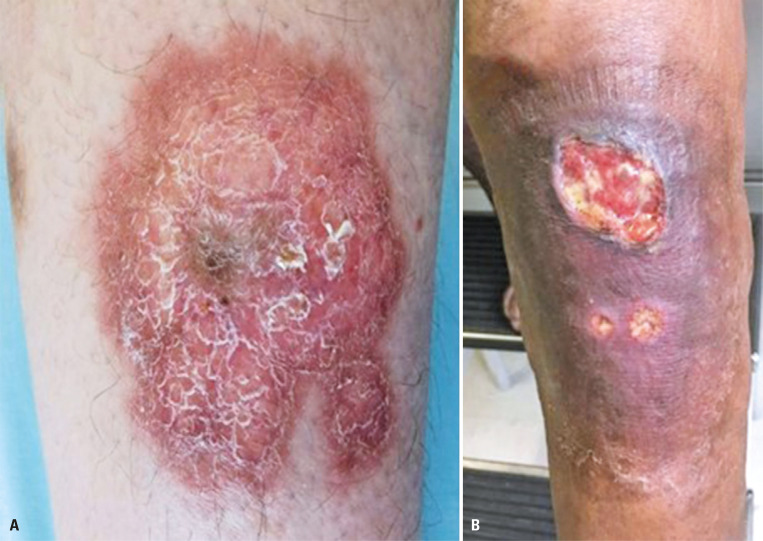
A) Erythematous and scaly plaque with a slight yellow tone on the center of the lesion, showing an initial form of necrobiosis lipoidica; B) Deep ulceration with violet erythema surrounding the lesion compatible with a late stage of necrobiosis lipoidica

Necrobiotic lipoidica (NL) is a rare idiopathic granulomatous disease of collagen degeneration with a risk of ulceration and is typically associated with DM.^21^ Its incidence in patients with DM is just 0.3 to 1.2 %; NL precedes DM in approximately 14% of patients, occurs simultaneously in up to 24%, and after DM in 62% of patients^22,23^ Due to its pathogenesis and association with autoimmune disease, NL has been demonstrated to be more associated with T1DM. One case report showed an association between HNF1A-MODY, NL, and granuloma annulare, highlighting the disease spectrum.^24^ Whilst patients with no other conditions can present with NL, the main correlated disorders are thyroid and inflammatory diseases, such as ulcerative colitis, rheumatoid arthritis, Crohn’s disease, and sarcoidosis.^25^ Usually, diagnosis of NL is usually difficult in the presence of other conditions based on the clinical presentation and associated locations, such as granuloma annulare, erythema nodosum, necrobiotic xanthogranuloma, chronic venous stasis ulcers, and sarcoidosis.^25^ Necrobiotic lipoidica has an age of onset between 30 and 40 years; females are likely to be more affected,^22^ and the role of genetics in disease pathogenesis remains unclear.^26^ Lesions are ordinarily bilateral and occur in the lower extremities, particularly in the pretibial area. Necrobiotic lipoidica usually starts with a slightly elevated and asymptomatic papule or nodule 1–3 mm in diameter, surrounded by sharp edges that corrode and can ulcerate in one-third of patients, evolving into well-demarcated yellow-brown plaques with violaceous borders, a central waxy atrophic appearance, and telangiectasias^25,27^ (Figure 2).

In darker skin, the plaques have a deeper yellow tone. An erythematous border was present around each stage of lesion development, and it may have several color gradations or be moderately erythematous despite not disappearing with pressure. Later, it may become round, oval, or even appear as irregular plates with well-defined edges, firm consistency, and a shiny surface. Ulceration may have appeared at the center. The surface was shiny, with yellowish spots, and crossed by thin vessels.^25,27^ A significant risk of secondary infections exists that can be aggravated by circulatory diseases with the appearance of shallow-bottom ulcerated lesions.^25,27^

No specific treatment exists for NL, although adequate glycemic control improves the prognosis and reduces the risk of secondary infections. Intradermal corticosteroid injections and surgical removal followed by grafting have already been attempted with conflicting results. Meticulous hygiene and antibiotics are recommended for treating active and/or secondary infections.^25,27^ The chronicity and occasional spontaneous remission of NL create difficulties in assessing the effectiveness of various proposed treatments.^25,27^

#### Bullosis diabeticorum

Bullosis diabeticorum (BD) is a rare condition in which spontaneous noninflammatory blisters appear in patients with DM.^28,29^ Bullosis diabeticorum usually occurs in patients with longstanding DM and affects 0.5% of patients in the United States, with a 2:1 male-to-female ratio.^30^ Its etiology remains unclear; however, its greater acral distribution suggests a role of vascular disorders and peripheral neuropathy in its pathophysiology.

Differential diagnosis from other bullous diseases, such as drug-induced skin reactions and bullous pemphigoid, must be considered through immunofluorescence, which is generally negative in this condition, and histopathological analyses.^28^ Its course is usually self-limiting, lasting approximately 2–6 weeks, and requires only supportive management. Secondary infections may require antibiotic therapy and surgical debridement.^29^

#### Scleredema diabeticorum

Scleredema Adultorum of Buschke (SAB) is an insidious connective tissue disease characterized by thickening and induration of the skin, with an erythematous and diffuse aspect, mainly affecting the face, back, shoulders, neck, and generally sparing the hands and feet.^31,32^ Scleredema Adultorum of Buschke is divided into three types, with type 1 being associated with chronic infections such as HIV and type 2 with rheumatological diseases and monoclonal gammopathy.^32^ Scleredema Adultorum of Buschke Type 3 is known as *scleredema diabeticorum* due to its association with DM (Figure 1). Prevalence varies between 2.5 and 14% among these patients, occurring mainly in those with longstanding DM, obesity, poor metabolic control, need for insulin, and chronic complications.^31,33^ The pathogenesis is not well understood, although it may be related to the glycation of collagen fibers, leading to altered degradation or stimulation of fibroblasts by hyperglycemia.^32^

The diagnosis is clinical, although histopathological confirmation may be needed to discard differential diagnoses such as mycosis fungoides and other infiltrating diseases.^33^ Progression of the disease is slow, and the prognosis is generally good, with spontaneous resolution within 18 months in approximately 50% of patients,^31^ although it can cause limited mobility and difficulties associated with insulin administration.^32^ The response to the available treatments is poor, and there is no clear correlation between glycemic control and prognosis.^31^ In this regard, various therapeutic modalities have been suggested, such as phototherapy, topical or systemic corticosteroids, and immunosuppressants, among others. However, these approaches are limited by a lack of consistent studies validating their benefits. Consequently, treatment must be tailored to individual patients.^34^ Psoralen and Ultraviolet A Phototherapy (PUVA) in patients with T1DM can improve symptoms and ameliorate insulin absorption in subcutaneous tissue, and the authors have been prescribing these therapeutic options with good results in these patients.^35^

#### Granuloma annulare

**Figure 3:**
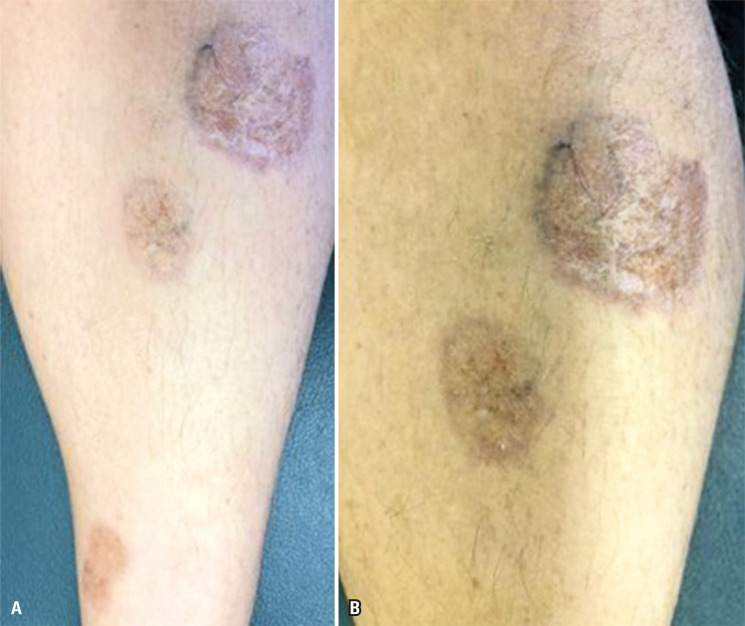
A and B: Brown, atrophic plaques in an annular pattern on the lower limbs, compatible with granuloma annulare

Granuloma annulare (GA) is a non-infectious granulomatous skin disease with variable presentations. Localized GA is the most common presentation and is characterized by flesh-colored to erythematous papules in annular configurations commonly observed on the dorsal hands or feet (Figure 3). Granuloma annulare is common in women under 30 years of age.^36^ Rings are usually less than 5 cm in diameter and can be enlarged by centrifugation. In the presence of ten or more lesions, the disease is considered generalized GA.

Subcutaneous GA is a rare form that affects children and adolescents and manifests as non-painful subcutaneous nodules. Usually, the clinical characteristics provide a diagnosis. However, in doubtful cases, a biopsy can confirm this, showing necrobiosis or mucinous degradation of collagen and either palisading or interstitial granulomatous infiltration.^36^ The association between DM and GA remains unclear. Although studies have shown an association between the two, others have failed to demonstrate such an association.^37-40^ Nevertheless, in patients with DM, GA appears to be more recurrent and chronic in patients with DM.^36^ Granuloma annulare often self-resolve, and patients frequently seek treatment.

Evidence regarding treatment is scarce, with a notable limitation that most studies are case series involving a small number of patients using topical medication. Local treatment is recommended as the first-line therapy in most patients, including topical corticosteroids or tacrolimus, destructive methods, and intralesional therapies. For generalized disease, immunomodulation with anti-malarial drugs or, in severe cases, immunosuppression with methotrexate, cyclosporine, or tumor necrosis factor α (TNF-α) inhibitors can be considered.^41^

#### Diabetic dermopathy

**Figure 4:**
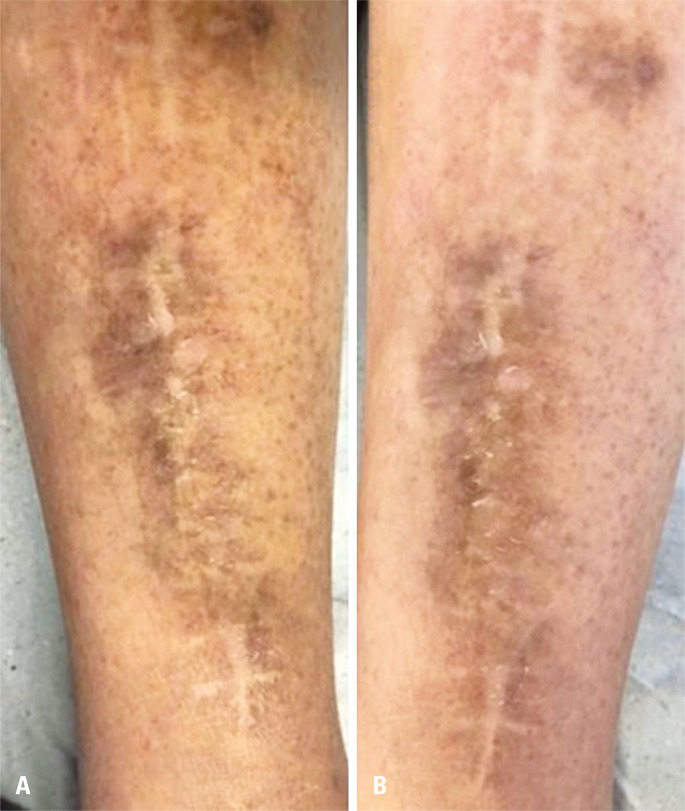
A and B: Hyperchromic diffuse macules on the lower limbs concentrated in trauma areas, compatible with diabetic dermopathy

Diabetic dermopathy is a prevalent clinical condition affecting up to 50% of patients with an essential clinical diagnosis. Although its pathogenesis remains controversial, there are indications that it is triggered by local trauma and microangiopathic changes.^42-45^ Diabetic dermopathy appears as pigmented pretibial spots^46,47^ predominantly bilaterally, as erythematous and rounded plaques or papules. In a few days, they evolve to a brownish tone, covered by fine scales^44^ (Figure 4). However, it can occur in less typical body areas, such as the forearms, thighs, and lateral malleoli, making the differential diagnosis of dermatophytosis important.^48^ In a few patients, this can be complicated by ulceration.

Men over 50 years of age with poor glycemic control are the most affected,^42^ with approximately three-quarters of elderly patients with DM having dermopathy. Dermopathy can precede the onset of DM, and it functions as a clinical marker for other chronic complications of DM and is associated with coronary artery disease, neuropathy, nephropathy, and retinopathy.^49,50^ No specific medical treatment is reported, just preventing secondary infectious complications. Periodic tracking of the possible underlying micro and macrovascular complications is recommended.^50^

#### Skin reactions due to device use

The current management of DM, particularly T1DM, often involves continuous glucose monitoring (CGM) and continuous subcutaneous insulin infusion (CSII) devices. These technologies can enhance glycemic control and treatment flexibility.^51,52^ However, the use of these devices entails the insertion of a small filament or cannula into the subcutaneous tissue and the application of an external adhesive patch, which can result in dermatological complications in 34–90% of users.^53-55^ Commonly reported skin lesions include erythema, lipodystrophy (specific to CSII use), subcutaneous nodules, scars, and pruritus.^53^

Despite the high incidence of dermatological issues, there is a paucity of evidence-based studies on managing skin integrity problems associated with these devices. Patients frequently resort to barrier creams, films, and additional patches through trial and error.^54^ Published practical orientations for skincare prophylaxis include avoiding application to broken skin or unhealed irritation (allowing one week for healing before reusing the area) and maintaining a distance of 1–2 inches from other infusion sets or sensors. Additional recommendations are to rotate through multiple sites, clean the skin with oil-free antimicrobial soap, ensure thorough drying, perform gentle exfoliation of oily skin, trim hair with a dry razor if necessary, and consider using unscented solids or spray antiperspirants to manage sweating-prone skin.^56^

In patients where the skin is compromised but not infected, over-the-counter symptomatic treatments may be used to alleviate pain and itching, with close monitoring for potential infections. If an infection is suspected, individuals are advised to consult their diabetes healthcare provider for evaluation and, if necessary, to prescribe antibiotics.^56^ The limitations of existing studies include reliance on self-reported data rather than clinical evaluation and a general lack of evidence-based guidance for managing skin integrity issues related to device use.

#### Diabetic foot ulcers

Up to 34% of patients with T1DM or T2DM will develop foot ulcers at some point in their lives, and approximately 20% of these ulcers will lead to amputation. The pathophysiology of this condition is multifactorial, resulting from sensory, motor, and autonomic neuropathy, leading to alterations in sensory, biomechanical, and skin integrity. Callus formation is common during the initial stages of these ulcers. Furthermore, the coexistence of peripheral arterial disease leads to reduced local blood flow, and the immune system impairment observed in patients with DM is a risk factor for the evolution of infected ulcers.^57-59^

Qualified healthcare professionals should conduct annual foot evaluations to screen for diabetic neuropathy and to detect vascular and skin changes. The evaluation should include medical history and physical examination assessing temperature or pinprick sensation (function of small fibers) and vibration sensation using a 128 Hz tuning fork (function of large fibers). In addition, a 10 g monofilament test should be performed annually to assess the absence of pressure sensation in at least three locations on each foot, defining the risk of ulceration and amputation.^57,60^ The “Wound, Ischemia, and Foot Infection classification system” was developed and validated to assess the risk of limb loss in patients with diabetic foot ulcers by combining three variables: wound, ischemia, and infection. This classification includes evaluation of the degree of tissue loss, ischemia, and foot infection as none, mild, moderate, or severe.^58,61^ The diagnosis of diabetic foot infection is predominantly clinical, as suggested by the presence of two or more inflammatory signs, such as erythema and edema, and findings, such as purulence, fluctuation, or lymphangitis. In patients with suspected osteomyelitis, radiography, erythrocyte sedimentation rate (ESR), probe-to-bone tests, and magnetic resonance imaging (MRI) can support the diagnosis.^58^ However, the gold standard for diagnosing osteomyelitis involves bone biopsy and culture. Early treatment of diabetic foot infections (with antimicrobials and/or surgical intervention) is necessary to reduce the risk of amputation. Peripheral arterial disease (PAD) can be assessed clinically (pulse palpation) or using arterial Doppler with measurement of the ankle-brachial index (ABI), where a value <0.9 has good diagnostic accuracy.^58,59,61^

Patients at low risk of ulcer development should be monitored annually. Patients at moderate and increased risk (two or more risk factors: loss of protective sensation, peripheral arterial disease, and foot deformity) should use individualized footwear and undergo regular evaluation by a podiatrist. Patients with PAD require specific vascular assessment and treatment.

Management of patients with foot ulcers may include several strategies. Debridement may involve the removal of necrotic tissue. Although the guidelines recommend debridement once or twice per week, there is a lack of randomized controlled trials to support this recommendation.^58^ A study of 154,644 patients with chronic wounds, including diabetic foot ulcers, showed that those receiving weekly debridement had a significantly higher healing rate (55% *versus* 28%, p<0.001) compared to those receiving less frequent debridement.^62^ The use of dressings can be used based on wound characteristics, such as location, presence, and/or degree of inflammation, and the amount of exudate.

Hyperbaric oxygen therapy can be an additional option for diabetic foot ulcers with peripheral arterial disease when standard treatment is ineffective. Two double-blind studies have reported improved wound healing, although the results are conflicting.^63,64^ Topical oxygen therapy may be considered in patients with treatment failure. A double-blind study involving 58 patients showed statistically significant results for complete wound healing after this therapy (p<0.05).^65^ Other publications, including a systematic review and pooled recent meta-analyses, showed improved healing with topical oxygen therapy compared to sham controls (43.0% *versus* 28.0%; RR=1.59; 95%CI=1.07–2.37; p=0.02).^66^ New approaches, such as topical fibrin and leukocyte-platelet patches, have shown promising results, but clinical study results are still varied.^67^

Finally, a multidisciplinary approach with specialized teams for diabetic foot care is crucial to significantly reduce the risk of lower limb amputations related to diabetic foot ulcers.^58^ In this regard, management of diabetic foot ulcers should be individualized and based on a comprehensive assessment of the patient’s needs, including risk factors, ulcer type and severity, and treatment response. Advances in therapies and implementing evidence-based practices can significantly improve patient outcomes.

#### Recurrent cutaneous infections in *diabetes mellitus*

Patients with DM have a higher risk of developing infections as well as a higher risk of mortality. This occurs because of risk factors, such as altered immune function, decreased leukocyte chemotaxis and phagocytosis, diabetic neuropathy, and altered circulation. This risk was higher in patients with poor metabolic control and higher HbA1c levels.^68,69^

Candidiasis is the most common fungal infection in DM and is mainly caused by *Candida albicans.* Clinical forms such as vulvovaginal, balanopreputial, and angular stomatitis stand out. Vulvovaginal and balanopreputial candidiases are the major causes of genital pruritus. Treatment can be challenging and consists of glycemic control and topical or systemic antifungal therapy.^68,70^ Pityriasis versicolor and extensive dermatophytosis, such as tinea corporis, are highly prevalent in patients with DM and are associated with microangiopathy and poor glycemic control. An important cause of fungal infections that correlate with diabetic foot, one of the most complex complications of DM, is onychomycosis and foot skin mycosis, caused mainly by *Trichophyton rubrum* and *Trichophyton interdigitale.*^71^

Bacterial infections associated with skin breakdown in patients with DM can vary and are alarming, such as those caused by *Staphylococcus sp.*, the most frequent pathogen, or *Pseudomonas sp.* The infection can be mild or severe and can manifest as boils, abscesses, or anthrax. Bullous erysipelas is more common among patients with DM, and its recurrence may occur; these patients should always be treated with systemic antibiotics.^70,72^

#### Other dermatoses associated with *diabetes mellitus*

One of the conditions associated with DM is eruptive xanthoma, which may manifest as obesity, chronic renal disease, and hypothyroidism. Moreover, it occurs when high levels of triglycerides (greater than 700mg/dL) are present, and metabolically uncontrolled DM can be a triggering factor. The lesions appeared as cup-shaped, isolated, or confluent yellow papules with erythematous bases. They can spread rapidly on the buttocks, elbows, and knees and may be associated with itching and pain. Eruptive xanthomas should be considered a serious condition because of the associated risk of pancreatitis.^70,73^ The treatment options include surgical removal, laser therapy, cryotherapy, and the management of serum triglyceride levels.^74^ Another condition associated with DM is *Rubeosis faciei,* which is characterized by facial and neck erythema, present in up to 60% of patients with DM, likely related to the loss of vasoconstrictor tone. Moreover, it typically reflects poor glycemic control and is associated with peripheral neuropathy.^75^ Strict glycemic control is the mainstay of treatment for this reddish complex.^76^

## FINAL CONSIDERATIONS

*Diabetes mellitus* is a highly prevalent disease associated with a range of dermatological manifestations. Therefore, knowledge and recognition of the most common dermatological lesions in these patients are essential for both endocrinologists and primary care physicians. Early recognition of these conditions by the attending physician is essential for initiating treatment and, when necessary, for referral to a dermatologist. Although each condition has a different pathophysiology and treatment, many will improve with better glycemic control; therefore, adequate management of *diabetes mellitus* is paramount.
